# Beyond Pandora's box: vast potential with significant challenges

**DOI:** 10.1093/fsr/owae017

**Published:** 2024-02-16

**Authors:** Qi Wang

**Affiliations:** Guangzhou Key Laboratory of Forensic Multi-Omics for Precision Identification, School of Forensic Medicine, Southern Medical University, Guangzhou, China

## Dear editors,

I recently read the insightful article by Dinis-Oliveira and Azevedo on the prospective role of ChatGPT and similar AI models in forensic science [1]. The article is an important guide to understanding the complex effects of integrating AI into this sensitive area. The authors highlight the great potential benefits of AI in forensic science but also caution about the ethical, legal, and technical challenges involved. I truly appreciate their detailed and thoughtful work on this subject. My considerations are expressed as follows:

1) The specialized nature of forensic science: forensic science, being inherently interdisciplinary, combines various fields such as biology, chemistry, and law [2]. This combination requires AI systems to be technically adept and to understand the context of forensic work. However, forensic science is not as widely known as other fields, leading to a lack of AI development focused on it. Most AI models are trained on more common data [3], so they might not grasp the complex details of forensic evidence. Therefore, it is crucial to direct AI research toward creating models that can accurately handle the specific challenges of forensic science.
2) Data limitations and privacy concerns: in forensic science, the development and effectiveness of AI systems are significantly limited by data constraints and privacy issues. Strict privacy laws and ethical considerations make it difficult to access a wide range of comprehensive data [4]. Forensic data, which include sensitive information such as DNA profiles and digital footprints, make it particularly challenging to create large, anonymized datasets for AI training. Furthermore, the diverse types of forensic evidence, ranging from biological samples to digital traces, demand a variety of data that are hard to gather while maintaining ethical standards.
3) Variability in legal and forensic frameworks: implementing AI in forensic science is made more complex due to the diverse legal and forensic frameworks across various regions. AI systems need to be flexible enough to meet different legal standards, criteria for accepting evidence, and procedural details specific to each jurisdiction. To develop AI models capable of handling this complexity, there is a need for technological advancements coupled with a thorough grasp of the legal and forensic processes unique to this field.
4) Technology adoption pace in forensic science: forensic science traditionally adopts new technologies at a cautious pace, prioritizing reliability and validity over speed [5]. This cautious approach is essential because mistakes in forensic analysis can lead to significant legal and personal consequences. Therefore, incorporating AI into forensic science requires thorough testing and validation to ensure that it meets the field’s strict standards.

Addressing the phenomenon of AI “Hallucinations” in forensic science: Additionally, it is imperative to address the phenomenon of “hallucinations” or the generation of inaccurate or fictional content by large language models. These AI-driven models, while highly advanced, do not possess true understanding or consciousness, leading to potential errors or fabrications in their outputs [6]. Alarmingly, these inaccuracies can be challenging to detect, even for professionals with relevant expertise. Sometimes, the falsifications are so convincing that they can deceive even established scientists in the field [7]. In forensic applications, where the accuracy of information is paramount, reliance on AI-generated content must be approached with extreme caution. The integration of these models into forensic processes necessitates a framework that not only leverages their capabilities but also incorporates rigorous verification measures by skilled forensic experts. This will ensure that the AI’s potential is harnessed effectively while safeguarding against the risks of misinformation, thereby maintaining the integrity and reliability of forensic investigations.

Moving forward: building a sustainable AI framework in forensic science:

1) Ethical and legal foundations: establishing robust ethical guidelines and legal frameworks is paramount. This involves not only ensuring the accuracy and fairness of AI models but also safeguarding privacy and handling data responsibly.
2) Comprehensive datasets: collaborative efforts are needed to compile extensive, anonymized datasets that can cater to the diverse needs of forensic science. This requires balancing the need for comprehensive data with the ethical considerations surrounding data collection and use.
3) Standardization and adaptability: the development of technical standards specific to forensic applications should go hand-in-hand with ensuring that AI models are adaptable to different legal and procedural contexts.
4) Stakeholder engagement: engaging a wide range of stakeholders, from forensic experts to legal professionals, in the development and implementation of AI in forensic science is crucial. This collaborative approach ensures that AI solutions are grounded in the realities of forensic work.

While the integration of AI in forensic science presents a complex array of challenges, it also offers a path to significant advancements in the field. A cautious yet forward-thinking approach is essential to navigate this journey successfully. We should be enthusiastic about the potential of AI in transforming forensic science and look forward to contributing to this evolving conversation. 
